# Claims of causality in health news: a randomised trial

**DOI:** 10.1186/s12916-019-1324-7

**Published:** 2019-05-16

**Authors:** Rachel C. Adams, Aimée Challenger, Luke Bratton, Jacky Boivin, Lewis Bott, Georgina Powell, Andy Williams, Christopher D. Chambers, Petroc Sumner

**Affiliations:** 1 Cardiff University Brain Research Imaging Centre (CUBRIC), School of Psychology, Cardiff University, Cardiff, UK; 2 School of Psychology, Cardiff University, Cardiff, UK; 3 School of Journalism, Media & Cultural Studies, Cardiff University, Cardiff, UK

**Keywords:** Science news, Science communication, Media, Public health

## Abstract

**Background:**

Misleading news claims can be detrimental to public health. We aimed to improve the alignment between causal claims and evidence, without losing news interest (counter to assumptions that news is not interested in communicating caution).

**Methods:**

We tested two interventions in press releases, which are the main sources for science and health news: (a) aligning the headlines and main causal claims with the underlying evidence (strong for experimental, cautious for correlational) and (b) inserting explicit statements/caveats about inferring causality. The ‘participants’ were press releases on health-related topics (*N* = 312; control = 89, claim alignment = 64, causality statement = 79, both = 80) from nine press offices (journals, universities, funders). Outcomes were news content (headlines, causal claims, caveats) in English-language international and national media (newspapers, websites, broadcast; *N* = 2257), news uptake (% press releases gaining news coverage) and feasibility (% press releases implementing cautious statements).

**Results:**

News headlines showed better alignment to evidence when press releases were aligned (intention-to-treat analysis (ITT) 56% vs 52%, OR = 1.2 to 1.9; as-treated analysis (AT) 60% vs 32%, OR = 1.3 to 4.4). News claims also followed press releases, significant only for AT (ITT 62% vs 60%, OR = 0.7 to 1.6; AT, 67% vs 39%, OR = 1.4 to 5.7). The same was true for causality statements/caveats (ITT 15% vs 10%, OR = 0.9 to 2.6; AT 20% vs 0%, OR 16 to 156). There was no evidence of lost news uptake for press releases with aligned headlines and claims (ITT 55% vs 55%, OR = 0.7 to 1.3, AT 58% vs 60%, OR = 0.7 to 1.7), or causality statements/caveats (ITT 53% vs 56%, OR = 0.8 to 1.0, AT 66% vs 52%, OR = 1.3 to 2.7). Feasibility was demonstrated by a spontaneous increase in cautious headlines, claims and caveats in press releases compared to the pre-trial period (OR = 1.01 to 2.6, 1.3 to 3.4, 1.1 to 26, respectively).

**Conclusions:**

News claims—even headlines—can become better aligned with evidence. Cautious claims and explicit caveats about correlational findings may penetrate into news without harming news interest. Findings from AT analysis are correlational and may not imply cause, although here the linking mechanism between press releases and news is known. ITT analysis was insensitive due to spontaneous adoption of interventions across conditions.

**Trial registration:**

ISRCTN10492618 (20 August 2015)

**Electronic supplementary material:**

The online version of this article (10.1186/s12916-019-1324-7) contains supplementary material, which is available to authorized users.

## Background

Each year, thousands of news stories make claims about health and millions of readers use them as their main source for up-to-date information [1–3]. Established news media are the most widespread means to disseminate beneficial information [4], but misleading claims are common [5, 6] and may damage public health and create confusion and mistrust [7–11]. The Academy of Medical Sciences recently reported that only 37% of British adults trust scientific evidence [12], potentially undermining the timely seeking of, and engagement with, medical or healthcare advice [13]. Trust entails that strong claims are backed by strong evidence and that caution and caveats are expressed where appropriate. But in a competitive media market, it is common to assume that news has no place for cautiousness and caveats. Here, we test this assumption.

Most biomedical and health news stories make a prominent causal claim in either the headline or first two sentences (e.g. ‘statins raise diabetes risk’; ‘statins slash breast cancer death rates’). It is these headlines and main claims that are most eye-catching, most shared and that also frame the rest of a story [14, 15]. However, many are based on correlational evidence [16, 17], where causal conclusions often prove incorrect [18]. For example, in a sample of 130 prominent health stories, 49% had causal claims based on non-randomised designs [6] (see also Additional file 1: Figure S1). Thus, our first intervention (described below) attempted to improve the alignment between the strength of prominent news claims and the nature of the underlying evidence.

Later in a news story, caveats occasionally appear, adding a qualification about the work. These are rare and normally nonspecific, such as suggesting more research is needed [19–21]. News almost never explicitly comments on whether the evidence can support a strong causal claim, such as mentioning the limitations of correlational data. Our second intervention attempted to change this.

Changes to science and health news are most likely to be achieved via press releases from journals, universities and funders, which stimulate and provide content for news. Previous observational research has found that health news content is strongly associated with press release content [5, 22–25]. Thus, we undertook a randomised trial intervening in press release content, moderating causal claims and inserting caveats in press releases as means to improve health news. The critical questions were whether news would change, whether the ability to attract news would drop and whether the suggested improvements would be feasible at scale.

## Methods

### Overview

In collaboration with nine UK press offices, we ran a randomised controlled trial in which the ‘participants’ were press releases (*N* = 312) distributed to international media outlets over a 20-month period from September 2016 to May 2017. To operationalise evidence strength, we concentrated on the basic distinction between correlational and experimental types of evidence, a keystone for assessing the ability to support causal conclusions [26].

The collaborating press offices sent their biomedical and health-related press releases to us just prior to release. We randomly allocated each press release to receive one, both or neither of two interventions. The first intervention was *causal claim alignment*. We made suggestions to align the headline and prominent claims with the evidence, such that direct causal claims were only made for experimental evidence, while correlational data carried cautious claims, using words such as *might* and *may*. The second intervention was a *causality statement/caveat*. We inserted an explicit statement about whether the evidence could support a causal conclusion (e.g. *this was an observational study, which does not allow us to conclude that drinking wine caused the increased cancer risk*).

The press office was then free to accept, edit or reject the proposals (sometimes in consultation with academics according to their normal procedures) and issued the release as normal. We searched for arising news (print, online and broadcast; total *N* = 2257), and its content was double-coded by two researchers blind to condition and press release content. The protocol was pre-registered (10.1186/ISRCTN10492618, 20/08/2015) and approved by the Research Ethics Committee at the School of Psychology, Cardiff University. We do not name press offices to avoid identifying individuals. All data are available online at https://osf.io/apc6d/

### Participants: press releases

**Fig. 1:**
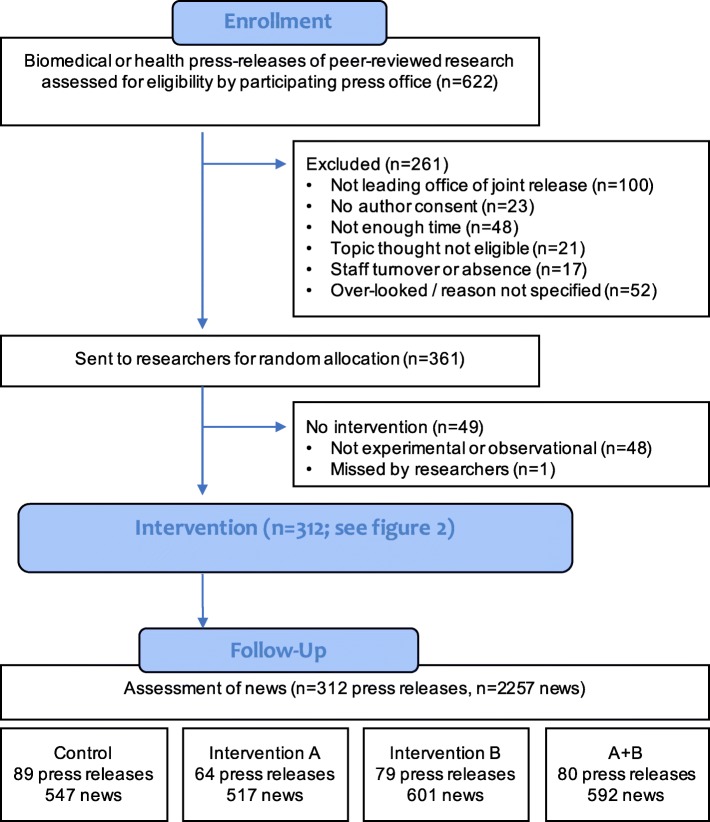
CONSORT diagram for the press releases (participants) in the trial. Inclusion criteria: participating press offices were asked to send each press release based on peer-reviewed research that was relevant to human health, broadly defined (all biomedical, psychological or lifestyle topics), where the press office was leading the press release (rather than collaborating on a release by another office outside the trial) and the academic authors consented (we used opt-out consent). Our focus was on observational and experimental studies. Observational studies included cross-sectional and longitudinal designs as well as meta-analyses and systematic reviews based solely on observational research. Experimental research included randomised controlled trials, other experiments and meta-analyses or systematic reviews based solely on experimental designs. Press releases on studies that could not be classified as experimental or observational (e.g. simulations and mixed methods reviews) were excluded

The ‘participants’ in the trial were press releases. For inclusion criteria, see Fig. 1.

### Sample size

We estimated we would achieve 300–500 press releases based on 100% coverage of eligible press releases from participating offices. In practice, some offices released fewer relevant press releases than expected and some eligible press releases were not sent to us for a variety of reasons (Fig. 1; 261 of 499 eligible press releases were sent; see reasons beyond the exclusion criteria of joint release and author consent). We therefore extended the trial duration and introduced a stopping rule of 75 press releases per bin (prior to exclusion of study designs not classifiable as experimental or correlational). Since we used pure randomisation, some bins were larger than others (Additional file 1: Table S2) and the total was 312 following study-design exclusion. Note that the power calculations in the protocol are only indications, since actual power depended on the clustering structure in the GEE analyses.

### Randomisation and blinding

Randomisation was by independent random number generation for each press release received (and therefore allowed unequal cell sizes by chance) and occurred prior to any assessment of content (and therefore before exclusion of simulations and mixed-methods reviews which reduced some cells below 75; Table 1). We did not communicate the condition to the press office. There were three researchers coordinating the trial (RCA, AC and LB). For each batch of press releases, RCA or AC coordinated randomisation and interventions, while the other two would remain blind for double-coding the outcomes.

**Table 1:** Numbers of press releases in each intervention condition following all exclusions, and numbers of intervention suggestions made and adopted

Intervention target	Intervention suggestion and uptake	Condition			
		Control	Causal claim alignment	Causality statement/caveat	Both interventions
	N	89	64	79	80
Causal claim alignment	Causal claim already aligned ^a^	-	25	-	30
	Alignment suggested and adopted ^b^	-	13	-	22
	Alignment suggested and not adopted ^b^	-	9	-	15
	No causal claim present ^c^	-	17	-	13
Statements/caveats about causality	Statement/caveat already present	-	-	27	21
	Statement/caveat suggested and adopted ^d^	-	-	31	35
	Statement/caveat suggested and not adopted	-	-	21	24

^a^ Both headline and main claim were already aligned to the evidence (where causal claims were made). No suggestions made

^b^ Suggestions were made only where causal claims existed and were not already aligned to the evidence. Alignment for the main claims was suggested in 59 press releases. Of these 22 suggestions were also made for the headline (9 adopted)

^c^ If no causal claim was present, suggestions could not be made. These press releases were still included in ITT analysis, but could not be included in AT analysis (where group allocation was based on alignment of causal claims made)

^d^ Causality statements were suggested for experimental evidence, and caveats were suggested for correlational evidence. These suggestions did not depend on the presence of causal claims in headlines or main claims. All press releases were entered into ITT and AT analyses

### Interventions

A. Causal claim alignment

The main causal claims in the headline and body of the press release were altered to align with the evidence underlying those claims. If claims were already aligned with the evidence, these were not modified. Based on previous results [27] showing which causal phrases readers distinguish or treat equivalently, all claims for observational evidence were modified to use hedged/cautious or associative language (*may*, *could*, *might*; e.g. ‘drinking wine may increase cancer risk’; *associated, linked*; e.g. ‘drinking wine is associated with increased cancer risk’) unless such language was already used. Claims for experimental evidence were modified to (or left as) direct causal statements (e.g. ‘drinking wine increases cancer risk’) or *can cause* statements (‘drinking wine can increase cancer risk’). In the registered protocol, we referred to *alignment* as *accuracy* (see Additional file 1: Figure S2).

B. Causality statement/caveat

Unless it already existed, a statement was inserted into the press release body to convey the design of the study and the strength of causal conclusions that could be justified from this design. For example, ‘this was an observational study, which does not allow us to conclude that drinking wine caused the increased cancer risk’ or ‘this study was a randomised controlled trial, which is one of the best ways for determining whether an intervention has a causal effect’ (in the registered protocol, we labelled this intervention *study design statement*; see Additional file 1: Figure S2). These statements were inserted at the earliest point where they fitted with the press release content. The majority were inserted into text, not into quotes, because feedback from press officers indicated that it was normally not pragmatic to get author approval for new quotes before release.

A. Causal claim alignment + causality statement

In this condition we suggested changes according to both A and B above, unless they were already present.

B. Control

The control condition was a suggested synonym change for a word that was not relevant to the main causal claims or study design (e.g. ‘beverage’ changed to ‘drink’).

### Primary outcomes

1. News content

From each pre-intervention press release, a list of search terms was generated to search for print, online and broadcast news coverage from a pre-defined list of top-tier national and international news outlets (see Additional file 1: Figure S3). Searches were conducted using Nexis, Google and TV Eyes. News coverage was sourced for 1 week prior to the press release date (to cover date differences due to time zones and any breaches of embargo) and for 28 days following the release. Two researchers blind to condition and final press release content coded the news using a standard protocol abbreviated from Sumner et al. [25] to extract the content outcomes listed below. All discrepancies in coding were resolved so that the final concordance was 100%. See open data for the full coding sheet.

- *Causal headline and claim alignment*: We coded whether the news headline and news main claims were *direct causal*, *can cause* or *hedged causal/associative.* Alignment was defined relative to the study design of the peer-reviewed journal article. Following Adams et al. (2017), we grouped *direct cause* and *can cause* together as *strong claims* appropriate for experimental evidence, and we refer to *hedged cause/associative* statements as *cautious claims* appropriate for correlational evidence [27]. We coded and analysed headlines and main claims separately as they are normally written by different people (sub-editor and journalist); headlines are most prominent but the writers are one step further removed from the press release. We operationalised main claims as those made in the first two sentences beyond the headline (excluding context sentences not about the new study). We excluded news headlines or claims that were not causal/associative or made a claim of no cause (‘wine does not raise cancer risk’). We also excluded news claims that were about entirely different variables than the press release.
- *Causality statement/caveat*: We coded whether a statement relating study design to cause-and-effect was present in news stories. We did not require that the news used scientific terms such as correlation or randomised controlled trial, but rather that the news contained a relevant statement about the possibility or difficulty of causal inference. For correlational evidence this had to be a caveat (e.g. ‘we don’t know if wine is directly responsible for cancer risk’ or ‘we cannot draw conclusions about cause and effect’).

2. News uptake

It is the proportion of press releases that attract news. Following Sumner et al. [20, 25], we simply scored news as present or absent, rather than discriminating between types of news and the differing media targets that some press releases may have. We also counted number of news stories (though this is an imperfect measure due to non-independence where some stories are copied across outlets; we present the results in Additional file 1: Figure S4). Although for news content we separated headlines and main claims, the outcome measure of uptake does not separate them. Therefore, we operationalised aligned press releases as follows: press releases for observational studies were aligned only if *both* headline and main claim used cautious language (and conversely, press releases for experimental studies were aligned if *either* the headline or the main claim used direct or *can cause* phrases).

### Secondary outcomes

We also coded whether news contained exaggerated advice or exaggerated inference from non-human research. These outcomes do not correspond to our main interest here, but were included for comparison with previous research [20, 25]. Analysis and results are in Additional file 1: Figure S5.

### Feasibility and acceptability

As a pre-requisite for interpreting the main news outcomes, and to assess whether alignment, caution and caveats are generally feasible and acceptable to integrate in press releases, we assessed the number of pre-intervention press releases that already contained them spontaneously, the number of suggestions made, accepted (including those edited while maintaining the distinction between cautious and strong), or rejected, and hence the numbers of our intended interventions present in the released versions of the press releases in each condition. Note that for our interest, spontaneous presence of appropriately cautious claims or caveats is more valuable than accepting our interventions, since intervention is not a feature of normal press release process. For this reason, we also assessed change between the trial and a baseline period of 2 years prior to the trial. To do this, we randomly sampled up to 20 press releases for each collaborating centre from 2014 and 2015 (10 from each year, or all eligible press releases from a centre if less than 10 were available), using the same eligibility criteria (except consent, as these press releases are in the public domain). We double-coded them in the same way as the press releases in the trial.

### Analysis and statistical methods

We focus the analysis on the main effects of causal claim alignment and causality statements/caveats separately, as recommended by [28], because the 2 × 2 design was not powered for the interaction (we report interactions as secondary analyses [28]). Causal phrasing could be coded and analysed where the headline or main statement made a causal or associative claim (excluding those that made no claim about a health outcome, or made a claim of no cause, e.g. *wine does not cause*…). Presence or absence of causality statements/caveats could be assessed for all. For causal claim alignment, we also separated news headlines and main claims, as explained above.

For the primary outcome measures of news content and uptake, we used both *intention-to-treat* (ITT) and *as-treated (AT)* analytic approaches. ITT analysis maintained the randomisation, comparing news content and uptake in conditions that attempted to make interventions against those that did not regardless of whether a suggestion was possible or accepted, and what the final press releases actually contained. AT analysis, on the other hand, depended on the content of the finally released press releases. This corresponds directly to what the journalists actually saw, but it disregards the randomisation and is therefore an associative analysis subject to selection bias, for which causal inference is not directly possible. However, it becomes useful when there are high levels of treatment mixing within groups due to spontaneous presence in the control group or non-acceptance in the intervention group—both of which we anticipated here and which can render ITT difficult to interpret (and would also severely reduce N for a *per-protocol* analysis, which we did not perform).

To account for the clustering of news to press releases or press releases to press office, we used generalised estimating equations (GEE, using a binary logistic model with exchangeable correlation matrix) as in our previous work [20, 25]. Since our intervention suggestions depended on study design (observational vs experimental), we also tested interactions with study design (data plotted in Additional file 1: Figure S6).

To assess feasibility, we estimated usage rates of caution and caveats in both pre-intervention and final press releases and compared them to the baseline period, using GEE as above to compensate for the clustering of press releases to press office.

## Results

### Causal headlines and main claims

#### News content

**Fig. 2:**
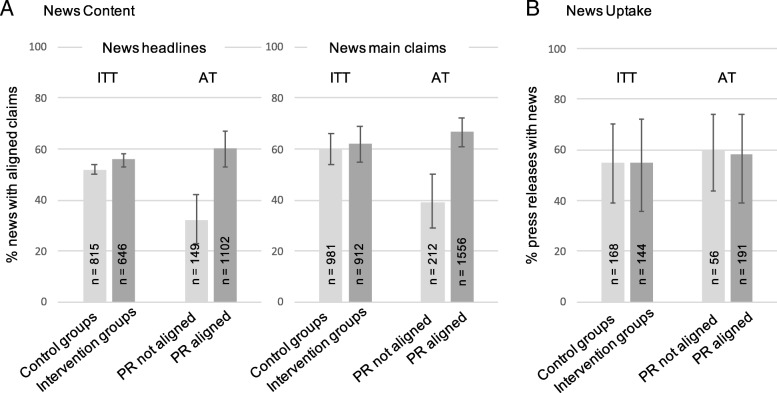
**a** News follows the phrasing of the press release: In ITT and AT analysis, news headlines were more likely to align to evidence if the press release phrasing did so; and in the AT analysis, claims in the news text were also more likely to do so if the press release did so. The discrepancy between ITT and AT analyses was due to a high level of condition mixing (see text). **b** ITT and AT analyses both show no evidence of reduced news uptake for press releases whose headlines and main claims aligned to evidence (see also Additional file 1: Figure S4 for the average number of news per press release). Error bars are 95% CIs. For each bar, *n* reports total number of news (**a**) or press releases (**b**) in that analysis group (i.e. the denominator of the proportion that the bar displays; total n is lower for AT than ITT analysis, because AT was possible only for press releases with causal claims present in headlines or main claims)

We coded whether the news headline and news main claims were *strongly causal* or *cautious*, following the distinctions readers make between causal phrases [27]*.* Alignment was defined as *strong claims* for experimental evidence and *cautious claims* for correlational evidence. We coded and analysed headlines separately from main claims in the body of the story as they are normally written by different people (sub-editor and journalist); headlines are most prominent but the writers are one step further removed from the press release. We used both *intention-to-treat* (ITT) and *as-treated (AT)* analytic approaches. For the ITT analysis, we compared news for the intervention groups where we intended to make suggestions for causal claim alignment (whether in isolation or combined with causality statements/caveats, and regardless of whether suggestions were accepted), with news from the press releases without alignment suggestions (regardless of whether alignment already existed in these control press releases). ITT analysis revealed a small significant rise the proportion of aligned news headlines for the groups with press release headline intervention compared to those without (Fig. 2a left; 56% [53 to 58] vs 52% [50 to 54], 95%CI of the OR = 1.2 to 1.9). The equivalent comparison for main claims was not significant (Fig. 2a right; 62% [55 to 69] vs 60% [54 to 66], 95% CI of the OR = 0.7 to 1.6). ITT analysis was relatively insensitive because the majority of control press releases also contained alignment through spontaneous adoption (see below). The interaction with causality statements (for which the study was not powered) was significant for headlines (OR = 1.3 to 2.2), such that the main effect was driven by the condition with both interventions (estimates for the conditions control, claim alignment, causality statement and both were 52%, 49%, 52% and 62%, respectively). The interaction was not significant for main claims (OR = 0.5 to 2.4; estimates for the conditions: 59%, 59%, 62% and 65%).

For AT analysis, we compared news for press releases that did or did not have aligned headlines and main claims at the point of release. This corresponds directly to what the journalists actually saw, but it disregards the randomisation and is therefore an associative analysis. This was possible for 247 press releases that contained a causal claim present in the headline or main claim. The proportion of news headlines using aligned language was 60% (CI 53 to 67%) when the press release headline did so, compared to 32% (CI 23 to 42%) when the press release did not (OR = 2.4, CI 1.3 to 4.4; N news = 1251). The proportion of news main claims using aligned language was 67% (CI 61 to 72%) when the press release did so, compared to 39% (CI 29 to 50%) when it did not (OR = 2.8, CI 1.4 to 5.7; N news = 1768). Note that the majority of the press releases were based on observational studies (72%; *N* = 179/247), where aligned claims meant cautious wording. These effects were still strong when analysing observational studies alone (headlines: 56 vs 23%, CI of the OR = 1.9 to 9.4; claims 64% vs 34%, CI of the OR = 1.8 to 6.4). The interactions with the study design were not significant (Additional file 1: Figure S6). Neither were the interactions with causality statements (headlines: estimates for the 4 cells were 31%, 65%, 33% and 54%, OR = 0.6 to 4.8; main claims: 41%, 71%, 37% and 62%, OR = 0.5 to 3.6).

#### News uptake

Importantly, there was no detectable cost to news uptake. The proportion of press releases that attracted news did not significantly differ in either ITT analysis (Fig. 2b; 55% vs 55%, OR = 0.7 to 1.3) or AT analysis (Fig. 2b; 60% vs 58%, OR = 0.7 to 1.7). The pattern was similar for observational and experimental studies with no significant interaction (see Additional file 1: Figure S7). The interaction with causality statements was underpowered and inconsistent across analyses: uptake for each cell (control, claim alignment, causality statement and both) was estimated as 54%, 60%, 57% and 50%, respectively in ITT (OR = 1.0 to 2.7), and 69%, 51%, 62% and 73%, respectively, in AT (OR = 1.5 to 8.2).

#### Feasibility/acceptability and group mixing

**Fig. 3:**
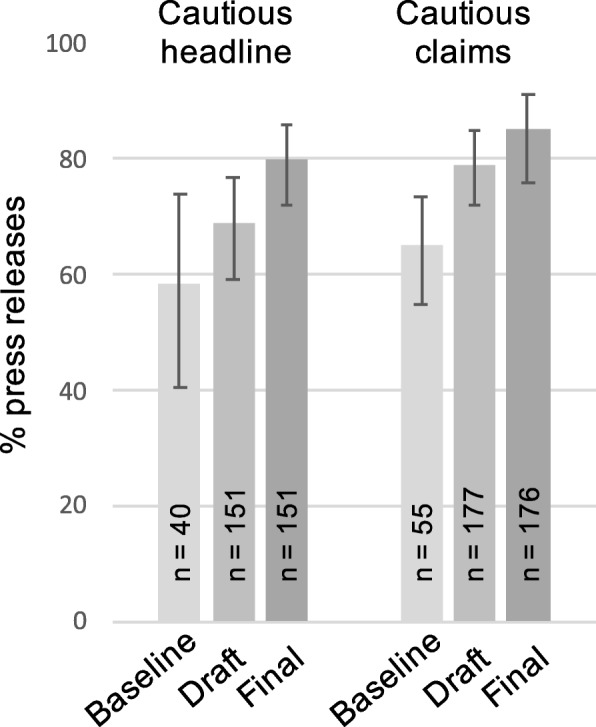
Feasibility and growing use of cautious headlines and main claims in observational research (error bars are 95% CIs). Feasibility is indicated by the increase in spontaneous use in pre-intervention (draft) press releases since the baseline period (2014/15). Final press releases showed small further increases in cautious wording following suggestions in the trial. For each bar, n reports the total number of press releases in that analysis group (i.e. the denominator of the proportion that the bar displays)

Since we already know that strong claims are common in press releases and news, the key interest was the feasibility of cautious claims for observational studies, employing words like *may*, *might* or using associative language. The majority of the press releases were based on observational research (73%; *N* = 229/312); among these, we could analyse 151 headlines and 177 main claims (excluding those that made no claim relating an IV and DV, or made a claim of no cause, e.g. *wine does not cause*….). Figure 3 shows the estimated proportions of headlines and main claims that were already cautious (i.e. aligned to their observational study design) in the pre-intervention text and in the final press releases, compared to the baseline period prior to the trial. The most salient point is the spontaneous increase in alignment in both headlines and main claims in pre-intervention press releases (mid-grey) since the baseline period (light grey; headlines OR = 1.6, 95% CI 1.01 to 2.6; main claims OR = 2.1, 95% CI 1.3 to 3.4). The further increase from pre-intervention to final press release followed suggestions in the relevant conditions of the trial. For headlines, in the subset where suggestions could be made, 41% were accepted (including those edited, but maintaining the distinction between cautious and strong); for the main claim, 60% were accepted.

Overall, cautious headlines and main claims occurred frequently in press releases of observational studies, demonstrating caution is feasible and acceptable to the authors. In most cases, this was already implemented in the draft press releases before any trial suggestions were made. This spontaneous presence of caution strongly indicates feasibility, but when added to incomplete intervention acceptance, it meant that the proportions of aligned claims in final press releases hardly differed across conditions (GEE estimates: with intervention 76% (56 to 88) of headlines and 91% (82 to 96) of main claims; without intervention 70% (61 to 78) of headlines and 82% (77 to 86) of main claims). This made ITT analysis much less sensitive than AT analysis.

### Causality statements/caveats

#### News content

**Fig. 4:**
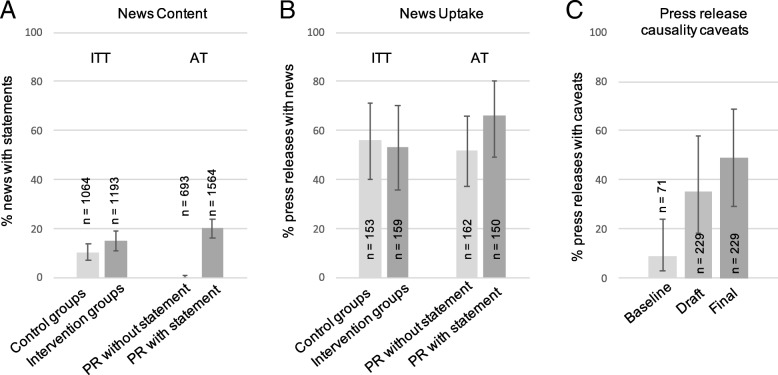
Use of causality statements/caveats (error bars are 95%CIs). **a** ITT was insensitive to differences in news content; AT showed that 20% of news contained causality statements or caveats if the press release did, and almost never otherwise. **b** ITT shows no reduction of news uptake and AT shows an increase in news for press releases containing causality statements/caveats (see also Additional file 1: Figure S4 for average number of news per press release). **c** Feasibility is indicated by the increase in spontaneous caveats for observational research since the baseline period (2014/2015). Final press releases showed a further increase following suggestions in relevant trial conditions. For each bar, n reports total number of news (**a**) or press releases (**b**, **c**) in that analysis group (i.e. the denominator of the proportion that the bar displays)

We coded whether a statement relating study design to cause-and-effect was present in news stories. We did not require that the news used scientific terms such as correlation or randomised controlled trial, but rather that the news contained a relevant statement about the possibility or difficulty of causal inference. For correlational evidence, this had to be a caveat (e.g. ‘we don’t know if wine is directly responsible for cancer risk’ or ‘we cannot draw conclusions about cause and effect’). ITT analysis found 15% (11% to 19%) of news contained causality statements for the conditions with statement/caveat suggestions, compared with 10% (7 to 14%) for the conditions without such suggestions (Fig. 4a, right, OR = 0.91 to 2.6). There was no interaction with claim alignment interventions (OR = 0.6 to 5.0, estimates for the four conditions were 8%, 12%, 16% and 14%).

AT analysis compared news for press releases with and without such statements/caveats regardless of the randomised condition, and found that the proportion of news containing a causality statement or caveat was 20% (CI: 16% to 24%) when the press release contained one compared to under 1% (CI: 0% to 1%) when it did not (OR = 50, CI: 16 to 156; N news = 2257). As noted above, the majority of these press releases were about observational studies where the causality statement was an explicit caveat. The effect was similarly strong in the observational studies alone (20% vs 1%, CI of the OR = 12 to 180) and did not interact significantly with study design (Additional file 1: Figure S8). There was an interaction with claim alignment (OR = 1.2 to 154, estimates for the four cells were 3%, 0%, 18% and 22%).

#### News uptake

ITT analysis showed no significant difference in news uptake between conditions with and without intervention (Fig. 4b left; 53% vs 56%, OR = 0.8 to 1.03). AT analysis showed higher news uptake for press releases containing causality statements/caveats (Fig. 4b right; AT 66% vs 52%, OR = 1.3 to 2.7). This effect was present in the observational studies alone for which these statements are explicit caveats (OR = 1.4 to 5.3) and did not interact significantly with study design (Additional file 1: Figure S8). Interaction with claim alignment is given in section A (the outcome measure of news uptake is identical here).

#### Feasibility/acceptability and condition mixing

The critical feasibility question concerns explicit caveats about causality for observational studies. Figure 4c shows that spontaneous usage of such caveats in press releases rose from under 10% in the baseline period (light grey, 2014/2015) to over 30% in the draft press releases in the trial (mid-grey, OR = 1.1 to 26). Following intervention in relevant trial conditions, 59% of suggestions were accepted, so that approximately half the press releases about observational studies contained explicit caveats about cause and effect when they were released (dark grey).

Spontaneous presence demonstrates feasibility, but meant there were causality statements/caveats in press releases in control conditions as well as intervention conditions (GEE estimates: with intervention 40% (30 to 51); without intervention 17% (7 to 36); OR = 1.7 to 6.6; these estimates differ in exact value from Fig. 4 because they include experimental and observational studies and because GEE adjusts estimates to different amounts given different clustering within press offices).

## Discussion

Prominent claims in news headlines and stories showed better alignment with the underlying evidence when press releases paid attention to this alignment. Additionally, 20% of news explicitly stated whether causality can be inferred when prompted to do so by press release text. Explicit causality statements have almost never been seen in news previously and almost never occurred in our large sample unless the press release contained it. Most of these statements were caveats and were not within quotes, making it more remarkable that they carried through to news (it is likely that carry-through for quotes would be higher). We found no evidence that news uptake is lower for press releases with aligned claims or caveats. The spontaneous use of explicit caution has risen since the baseline period before the trial, demonstrating that press officers find cautious headlines and explicit caveats feasible.

This trial was the first to intervene systematically in press release content and test the outcomes for health news. The main limitation was reliance on the AT (associative) analyses for most of the inferred effects of press release content on news content. The ITT analyses were insensitive and only significant for the effect on news headlines. The likely reason is that the trial saw a spontaneously increased rate of alignment in draft press releases before allocation to the condition—similar to a classic Hawthorne effect, but possibly because press offices that joined the trial were already changing their practices. For the narrow purpose of running a trial, this meant insufficient difference between conditions for sensitive ITT analysis. From a broader perspective, it is a strength that press officers have already demonstrated spontaneous willingness to apply the alignment and cautious language our interventions were suggesting. The pitfall for previous advice and guidelines for responsible science reporting has always been whether press officers and journalists find such guidance feasible within the constraints of writing pithy newsworthy text.

There are weaknesses and strengths for basing conclusions on AT analysis. It is correlational observation (although in this case, we know the linking mechanism between variables: journalists read the press releases). However, while ITT focusses on whether the *intervention protocol* itself causes a difference (with non-adherence to the protocol being an important part of the assessment), here, an intervention is not a normal part of the press release process. AT focuses on the content of the issued press releases. For this reason, it is more sensitive when assessing potential harms (in this case, the possibility of lower news uptake).

That news claims and causality statements/caveats correlated strongly with press release content (Figs. 2a and 4a) confirms previously observed associations for other content [5, 20, 24, 25, 29]. We built on this research in three main ways: previous findings have been based on naturally arising content, while we ran an intervention trial; we emphasised the key role of the headline (the most prominent and most difficult-to-influence part of a news story); our suggested in-text caveats were considerably more explicit than normally contained in news or press releases [20].

Readers are not expected to understand the technical distinctions between study designs that underlie stronger or weaker evidence. Indeed studies show that even college students who have taken research design courses find this difficult to discern [30–32]. What readers do perceive are systematic differences between levels of caution or strength in causal claims [27] and additional phrasing that implies caution [21] (‘One limitation…’). We focussed on these phrases that readers understand and differentiate.

### Unanswered questions

For our study, the outcomes were focussed on aligning prominent claims in news with underlying evidence. The extent to which this would influence public health is difficult to determine. Previous research has shown an association between health behaviour and specific topics in health news (e.g. vaccines, statins) [10, 11], and ‘spin’ in news has been experimentally shown to influence clinicians’ interpretation [33]. The effects of ubiquitously boosting the alignment between news and evidence remain to be tested. We would predict that better alignment could help achieve goals promoted by health academies: for example, reducing perceived conflict in health news and improving trust in evidence-based medicine (e.g. [12]). It could help readers make more informed health decisions and ultimately improve public health.

We limited our focus to only one facet of evidence strength, the distinction between experimental and correlational evidence, because of their fundamentally different relationship with causal inference [26]. Distinctions within these classes of design are just as important—such as between small-scale simple correlations and large epidemiological studies. Since our data showed similar patterns across study designs (Additional file 1: Figure S6, S7 and S8), we infer that the salient dimension for journalists is the confidence or caution in the claims, rather than the study design itself. Thus, our conclusions should apply to using cautious claims and caveats wherever relevant, transferring to other facets of evidence strength. This remains to be confirmed.

One unexpected result was the higher news uptake we found for press releases with caveats (Fig. 4b and Additional file 1: Figure S8B). Future research could test whether these explicit caveats increased perceived credibility [34]. Parallel research has found that caveats lead readers to rate researchers as less confident, without lowering interest [21].

## Conclusions

Our results imply that small changes in press release headline and claim wording, followed by explicit caveats or statements in the text, are a realistic means to improve coherence between the linguistic forcefulness of news claims and the evidence underlying those claims. Clinicians, scientists and press officers can take encouragement that deft caution and clear caveats are unlikely to harm news interest and can penetrate through to news and even to news headlines. If writers of abstracts, press releases and news were to systematically align cautious language (e.g. *may cause*) to most correlational evidence (unless the weight of evidence is unusually large), and strong language (direct constructions or *can cause)* to most experimental evidence (unless the weight of evidence is low), this would not only supply information to those who know how to interpret the convention, it would also cement a relevant and meaningful distinction for non-experts reading health and science news. Critically, this convention is pragmatic, as shown by the rates of spontaneous adoption (Fig. 3), making use of the phrases already used by writers and understood by readers. Equally importantly, this information can be carried by the headlines and prominent claims themselves, which most widely circulate via social media.

## Additional files

Additional file 1: **Figures** and **Tables S1** to **S8:** supplementary data, methodological detail and figures separating the data by study design. (PDF 219 kb)

## Abbreviations

AT: as-treated analysis
CI: Confidence interval
DV: Dependent variable
GEE: Generalised estimating equations
ITT: Intention-to-treat analysis
IV: Independent variable
N: Number
OR: Odds ratio

## References

[1] Castell S, Charlton A, Clemence M, Pettigrew N, Pope S, Quigley A (2015). Public attitudes to science. Ipsos Mori report for Department for Business Innovation and Skills.

[2] Schwitzer G (2015). Trying to drink from a fire hose: too much of the wrong kind of health care news. Trends Pharmacol Sci.

[3] Briggs CL, Hallin DC (2016). Making health public: how news coverage is remaking media, medicine, and contemporary life.

[4] Stryker JE, Moriarty CM, Jensen JD (2008). Effects of newspaper coverage on public knowledge about modifiable cancer risks. Health Commun.

[5] Yavchitz A, Boutron I, Bafeta A, Marroun I, Charles P, Mantz J (2012). Misrepresentation of randomized controlled trials in press releases and news coverage: a cohort study. PLoS Med.

[6] Haneef R, Lazarus C, Ravaud P, Yavchitz A, Boutron I (2015). Interpretation of results of studies evaluating an intervention highlighted in Google health news: a cross-sectional study of news. Courvoisier DS, editor. PloS one. Public Libr Sci.

[7] Grilli R, Ramsay C, Minozzi S (2002). Mass media interventions: effects on health services utilisation. CochraneDatabase Syst Rev.

[8] Sharma V, Dowd MD, Swanson DS, Slaughter AJ, Simon SD (2003). Influence of the news media on diagnostic testing in the emergency department. Arch Pediatr Adolesc Med.

[9] Schwitzer G (2008). How do US journalists cover treatments, tests, products, and procedures? An evaluation of 500 stories. PLoS Med.

[10] Ramsay ME (2013). Measles: the legacy of low vaccine coverage. Arch Dis Child.

[11] Matthews A, Herrett E, Gasparrini A, Van Staa T, Goldacre B, Smeeth L (2016). Impact of statin related media coverage on use of statins: interrupted time series analysis with UK primary care data. BMJ.

[12] The Academy of Medical Sciences (2017). Enhancing the use of scientific evidence to judge the potential benefits and harms of medicines.

[13] Boivin J, Bunting L, Koert E, Ieng UC, Verhaak C (2017). Perceived challenges of working in a fertility clinic: a qualitative analysis of work stressors and difficulties working with patients. Hum Reprod.

[14] Bransford JD, Johnson MK (1972). Contextual prerequisites for understanding: some investigations of comprehension and recall. J Verbal Learn Verbal Behav.

[15] Wiley J, Rayner K (2000). Effects of titles on the processing of text and lexically ambiguous words: evidence from eye movements. Mem Cognit.

[16] Haber N, Smith ER, Moscoe E, Andrews K, Audy R, Bell W (2018). Causal language and strength of inference in academic and media articles shared in social media (CLAIMS): a systematic review. Dorta-González P, editor. PloS one. Public Libr Sci.

[17] Wang MTM, Bolland MJ, Gamble G, Grey A (2015). Media coverage, journal press releases and editorials associated with randomized and observational studies in high-impact medical journals: a cohort study. Isales CM, editor. PloS one.

[18] Dumas-Mallet E, Smith A, Boraud T, Gonon F (2017). Poor replication validity of biomedical association studies reported by newspapers. Wicherts JM, editor. PloS one.

[19] Wang MTM, Bolland MJ, Grey A (2015). Reporting of limitations of observational research. JAMA Intern Med.

[20] Sumner P, Vivian-Griffiths S, Boivin J, Williams A, Bott L, Adams R (2016). Exaggerations and caveats in press releases and health-related science news. Wilsdon J, editor. PloS one.

[21] Bott L, Bratton L, Diaconu B, Adams RC, Challenger A, Boivin J, Williams A, Sumner P. Caveats in science-based news stories communicate caution without lowering interest. JEP Applied. 2019; in press. 10.1037/xap0000232 31246056

[22] Lewis J, Williams A, Franklin B (2008). A compromised fourth estate?. Journal Stud.

[23] Jackson D, Moloney K (2015). Inside Churnalism. J Stud Routledge.

[24] Schwartz L. M., Woloshin S., Andrews A., Stukel T. A. (2012). Influence of medical journal press releases on the quality of associated newspaper coverage: retrospective cohort study. BMJ.

[25] Sumner P., Vivian-Griffiths S., Boivin J., Williams A., Venetis C. A., Davies A., Ogden J., Whelan L., Hughes B., Dalton B., Boy F., Chambers C. D. (2014). The association between exaggeration in health related science news and academic press releases: retrospective observational study. BMJ.

[26] Atkins D, Best D, Briss PA, Eccles M, Falck-Ytter Y, Flottorp S (2004). Grading quality of evidence and strength of recommendations. BMJ.

[27] Adams RC, Sumner P, Vivian-Griffiths S, Barrington A, Williams A, Boivin J (2017). How readers understand causal and correlational expressions used in news headlines. J Exp Psychol.

[28] Montgomery AA, Peters TJ, Little P (2003). Design, analysis and presentation of factorial randomised controlled trials. BMC Medical Research Methodology 2003 3:1. Fourth. BioMed Central.

[29] Schat J, Bossema FG, Nederlands MN (2018). Overdreven gezondheidsnieuws. Relatie tussen overdrijving in academische persberichten en in nieuwsmedia. openaccess.leidenuniv.nl.

[30] Norris SP, Phillips LM, Korpan CA (2016). University students’ interpretation of media reports of science and its relationship to background knowledge, interest, and reading difficulty. Public Underst Sci.

[31] Mueller JF, Coon HM. Undergraduates’ ability to recognize correlational and causal language before and after explicit instruction. Teaching of Psychology. 9 ed. SAGE PublicationsSage CA: Los Angeles, CA; 2013;40:288–293.

[32] Bleske-Rechek A, Morrison KM, Heidtke LD (2014). Causal inference from descriptions of experimental and non-experimental research: public understanding of correlation-versus-causation. J Gen Psychol.

[33] Boutron I, Altman DG, Clinical SHJO (2014). Impact of spin in the abstracts of articles reporting results of randomized controlled trials in the field of cancer: the SPIIN randomized controlled trial. focusoptekst.nl.

[34] Jensen JD (2008). Scientific uncertainty in news coverage of Cancer research: effects of hedging on scientists and journalists credibility. Hum Commun Res.

