# Supplementary material for: Claims of causality in health news: a randomised trial
Source: BMC Med. 2019 May 16;17:91. doi: 10.1186/s12916-019-1324-7 (PMC6521363; doi:10.1186/s12916-019-1324-7)
Supplement: Supplementary file 1 — Figures and Tables S1 to S8: supplementary data, methodological detail and figures separating the data by study design. (PDF 219 kb) [file 12916_2019_1324_MOESM1_ESM.pdf]

Additional file 1:

Supplementary information for *Claims of causality in health news: a randomised trial.*

S1. Causal claims in health news in 2011

The main claims in 1250 health-related news stories arising from research in 20 leading UK universities [25] and 8 leading biomedical journals [20] in 2011 were coded independently by two researchers for the 3 levels of causal strength that readers are known to discriminate [27]: 1. *directly causal*, using words like *cuts, boosts, harms*; 2. *can phrases*, such as *can harm*, *can boost*; 3. a cluster of more cautious hedged and associative phrases, such as *may harm, might harm*, *associated with, linked to*. We also assessed whether the underlying peer-reviewed research was experimental or correlational. Exclusion criteria were: no causal/associative claim; simulations; qualitative research; mixed methods; leaving 906 news stories. Figure S1 shows that strong causal claims were almost as likely to be based on correlational evidence as on experimental evidence (panel A right bars). If readers intuitively infer stronger claims mean stronger evidence, they would be frequently misled. A binary logistic regression using causal phrase to attempt to predict the type of evidence achieved only 58% classification accuracy, compared to 54% chance level (because of uneven bin sizes; Regression b=0.98, S.E.=0.15; exp(b)=2.7, 95%CI 2.0 to 3.6). We also checked whether distinguishing between strictly associative claims (e.g. *associated with*) and conditional causal claims (e.g. *may boost*) aided classification, even though readers treat these phrases similarly [27]. It did not because the classification boundary still fell between *may cause* and *can cause.*

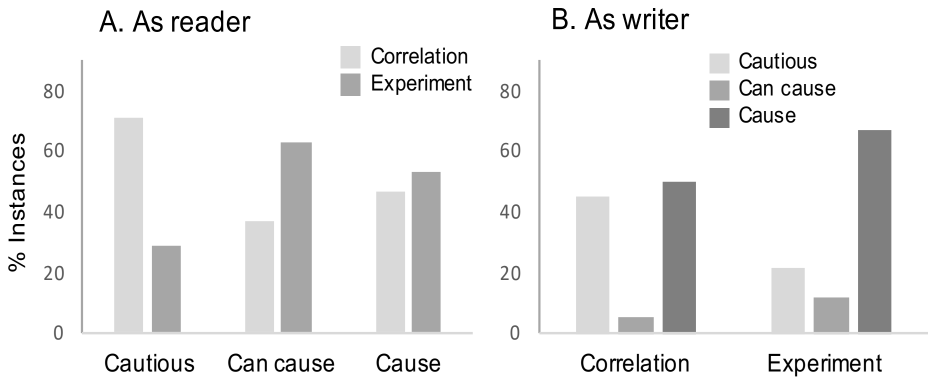

Figure S1. Panel **A** shows whether evidence was experimental or correlational from the point of view of *readers* encountering different strengths of causal claims in news. For example, of the 309 news claims with cautious causal phrases (left two bars), 71% were based on correlational evidence (left light bar) while 29% were based on experimental evidence (left dark bar); of the 522 news claims using direct causal expressions (right bars), 47% arose from correlational evidence (right light bar) and 53% arose from experimental evidence (right dark bar). In other words, when strong causal claims occurred, there were nearly as likely to have been based on correlational evidence as experimental. Panel **B** shows whether *writers* dealing with each type of evidence used cautious or strong claims. For example, for the 490 news claims based on correlational evidence (left three bars), 45% used associative or weak causal expressions, 6% used *can cause* expressions and 50% used direct causal expressions.

S2. Notes on the registered protocol

Title. The registration title was *Randomised controlled trial of optimal press release wording on health-related news coverage* We changed the title for the report because *optimal* was overstated – we test just two aspects of wording.

Intervention and outcome labels. In the protocol we used the word *accuracy* to refer to the alignment between causal claims and evidence. In the report we prefer *alignment* because it is not *inaccurate* to use a cautious phrase to refer to experimental evidence (instead it would simply not help distinguish evidence types).

In the registered protocol we used *design information* to refer to our suggested causality statements/caveats. The suggested statements or caveats always linked study design to causality, but we prefer to call them *causality statements/caveats* in the report for two reasons: 1. Our trial focused on relatively few aspects of study design, and 2. As an outcome measure in news, the critical feature was whether the statement/caveat mentioned causality, not whether it mentioned study designs. For example, 'we don't know if wine is directly responsible for cancer risk' would be sufficient to code a caveat as present, but 'in an observational study researchers found….' would not be sufficient to code a caveat as present.

News number and length. In the main report we simply present *percentage of press releases with news* as the uptake measure, following Sumner et al. (2014, 2016). In the protocol we list *the number and length of news articles* as measures of news uptake. However, number of news is problematic because when many articles arise, it is often the case that a large subset of them are nearly identical across media distributers. It was not specified in the protocol whether these should be counted individually or as a single news story. We present the results below (S4) counting all stories individually regardless of content overlap, as an approximation for news reach. We did not attempt to analyse news length, since early discussion with press officers indicated that it would be uninterpretable because the most prominent news outlets often have the fewest words.

Sample size. We estimated we would achieve 300-500 press releases based on 100% coverage of eligible press releases from participating offices. In practice some offices released fewer relevant press releases than expected and some eligible press releases were not sent to us for a variety of reasons (Figure 1; 261 of 499 eligible press releases were sent; see reasons beyond the exclusion criteria of joint release and author consent). We therefore extended the trial duration and introduced a stopping rule of 75 press releases per bin (prior to exclusion of study designs not classifiable as experimental or correlational). Since we used pure randomization, some bins were larger than others (Table S2) and the total was 312 following study-design exclusion. Note that the power calculations in the protocol are only indications, since actual power depended on the clustering structure in the GEE analyses.

Analysis. The registered protocol did not contain an analysis plan. We therefore followed our previous precedent of using GEE, combined with the logic dictated by our interventions and outcome measures, as explained in the report.

S3. UK and International media outlets used for news search.

UK	International	Broadcast	Online
The Daily Telegraph	Boston Globe (USA)	BBC 1	ap.org
Financial Times	Los Angeles Times (USA)	BBC 2	arabnews.com
International NY Times	New York Post (USA)	BBC Radio 1	bangkokpost.com
The Guardian	The Wall Street Journal (USA)	BBC Radio 2	bbc.co.uk
The Independent	Washington Post, The (USA)	BBC Radio 4 FM	bostonglobe.com
The Times	Boston Globe Sunday (USA)	BBC Radio 5 Live	businesstimes.com.sg
The Wall St. Journal Europe	Washington Post Sunday (USA)	BBC World Service	chicagotribune.com
City A.M.	Times of India, The (New Delhi)	BBC World News	chinadaily.com.cn
The Times Scotland	Moscow Times (Russia)	Bloomberg Television	chinapost.com.tw
The Guardian	Dominion Post (New Zealand)	CNN	cityam.com
Daily Telegraph Scotland	Age, The (Australia)	Channel 4	couriermail.com.au
Independent i	Financial Times (Europe)	Channel 5	dailymail.co.uk
The Times Ireland	New York Times, The (USA)	ITV1	dailyrecord.co.uk
Daily Express	New York Times Sunday (USA)	Sky News	dailystar.co.uk
Daily Mail	Hindu Business Line, The	BBC News	economictimes.indiatimes.com
Evening Standard (London)	Globe & Mail (Canada)	BBC 1 London	economist.com
Metro	The Australian	BBC Parliament	europe.chinadaily.com.cn
Morning Star	New Zealand Herald		express.co.uk
Weekly News, The	The Wall Street Journal (Asia)		ft.com
Scottish Daily Mail	China Daily Hong Kong (China)		gulfnews.com
Scottish Daily Express	Bangkok Post (Thailand)		heraldsun.com.au
Irish Daily Mail	Herald on Sunday (New Zealand)		hindustantimes.com
Midweek Sport	Financial Times (USA)		independent.co.uk
Daily Star	Financial Times (Asia)		indy100.independent.co.uk
The Sun	Financial Times (Middle East)		irishmirror.ie
Daily Mirror	China Daily Europe		khaleejtimes.com
The Scottish Sun	Hindustan Times (India)		latimes.com.
Scottish Daily Mirror	Straits Times, The (Singapore)		livemint.com
Daily Star Scotland	Nation, The (Thailand)		metro.co.uk
Irish Daily Mirror	Jakarta Post (Indonesia)		mirror.co.uk
Daily Mirror N. Ireland	Business Times, The (Singapore)		morningstaronline.co.uk
The Irish Sun	Star (Malaysia)		mydigitalfc.com
Weekend Sport	Today (Singapore)		nationalpost.com
The Sun Northern Ireland	South China Morning Post		nationmultimedia.com
The Independent on Sunday	National, The (UAE)		newscientist.com
The Observer	China Post (Taiwan)		nypost.com
The Sunday Telegraph	Khaleej Times (UAE)		nytimes.com
The Sunday Times	Herald Sun (Australia)		nzherald.co.nz
The Sunday Times Scotland	Sunday Herald Sun (Australia)		pressassociation.com
The Observer North	Sunday Age - M (Australia)		racingpost.com
Sunday Telegraph Scotland	Sun-Herald (Australia)		scmp.com
The Sunday Times Ireland	Mint (India)		scotsman.com
The Sunday Times N. Ireland	Arab News (Saudi Arabia)		sfgate.com
The Mail on Sunday	Times of Oman (Oman)		shanghaidaily.com
Sunday Express	Sydney Morning Herald		smh.com.au
Scottish Mail on Sunday	National Post (Canada)		standard.co.uk
Scottish Sunday Express	Shanghai Daily (China)		straitstimes.com
Irish Mail on Sunday	Gulf News (UAE)		sundaypost.com
Sunday Mirror	Financial Chronicle (India)		sundaysport.com
The People	Economic Times (India)		telegraph.co.uk
Sunday Sport	Chicago Tribune (USA)		theage.com.au
Daily Star Sunday	San Francisco Chronicle (USA)		theaustralian.com.au
Scottish Sunday Mirror	Hindu, The (India)		thedpost.com
Daily Star Sunday Scotland	Courier Mail (Australia)		theglobeandmail.com
The People Scotland			theguardian.com
Irish Sunday Mirror			thehindu.com
			thehindubusinessline.com
			thejakartapost.com
			themoscowtimes.com
			thenational.ae
			thescottishsun.co.uk
			thestar.com.my
			thesun.co.uk
			thesun.ie
			thesundaytimes.co.uk
			thetimes.co.uk
			timesofindia.com
			timesofoman.com
			todayonline.com
			uk.reuters.com
			washingtonpost.com
			wsj.com

S4. Average number of news stories per press release

News uptake or reach can be assessed in two main ways. In the main report we followed previous work assessing news uptake as % press releases with news (i.e the binary question: *did the press release gain any news or not?* Figures 2B and 4B). We can also assess the number of news per press release. The latter measure is problematic because many news stories are non-independent (they can be very similar or even copies of each other). Nevertheless, there is potential information in a measure of news reach that goes beyond the binary question. Figure S4 shows the pattern of results for average news number is highly consistent with the binary assessment of news uptake as plotted in Figures 2B and 4B.

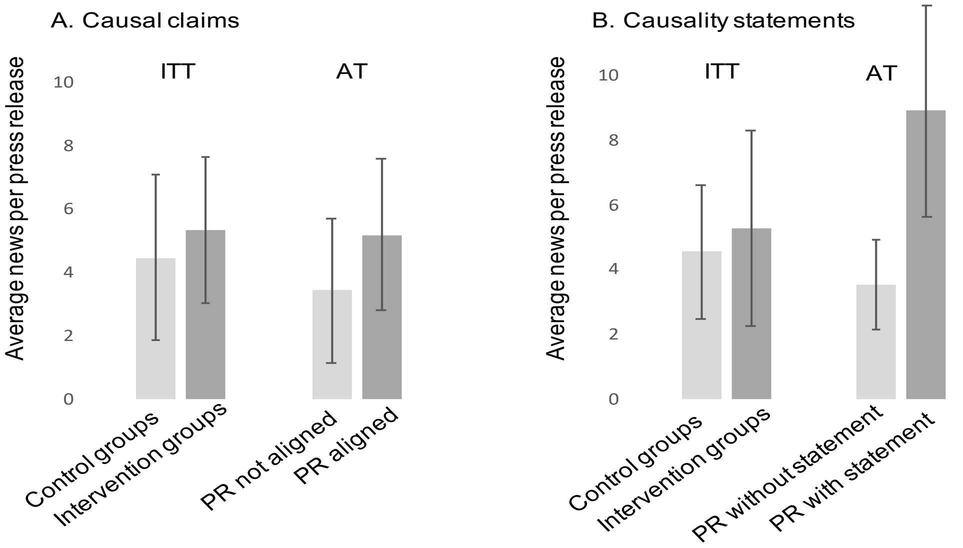

Figure S4. **A)** ITT and AT analyses both show no evidence of reduced numbers of news articles for press releases whose headlines and claims aligned to evidence. Error bars are 95%CIs. The AT analysis showed a significant increase in news (GEE, using a linear model with exchangeable correlation matrix, Exp(B)=0.17, 95%CI=0.06 to 0.47). **B)** ITT and AT analyses both show no evidence of reduced numbers of news articles for press releases with statements about causality. Again, the AT analysis showed a significant increase in news (Exp(B)=0.01, 95%CI=0.001 to 0.15). The number of press releases (denominator) for each bar can be found in Figures 2B and 4B.

S5. Advice to readers and claims about non-human studies

Advice. For comparison with Sumner et al. (2014, 2016), we analysed journal articles, press releases and news articles that contained at least one explicit advice statement anywhere in the text. We focused on direct advice that did not appear in the peer-reviewed journal article (*exaggerated advice* in Sumner et al, 2014). Rates of such advice were similar across press releases (22%) and news articles (27%). The odds of finding such advice in news was 34 times higher (p<.001; 95% CI: 9.1 to 127.26) when the press release contained it (81%; 95% CI: 57% to 93%) compared to when it did not (11%; 95% CI: 6% to 19%), replicating the previous research.

Human claims from non-human studies. For comparison with Sumner et al. (2014, 2016), we analysed the press releases and news arising from studies on non-humans. We focused on whether news and press releases made claims about humans that were not claimed in the peer-reviewed journal article. Human claims from non-human samples was very low across both press releases (0.5%) and news articles (2.2%). This may reflect increased willingness to openly discuss animal research than in previous years. The odds of such exaggeration in the news was 143 times higher (p<.001; 95% CI: 22 to 912) when the press release was similarly exaggerated (83%; 95% CI: 47% to 97% versus 3%; 95% CI: 2% to 6%).

S6. Causal headlines and claims by study design.

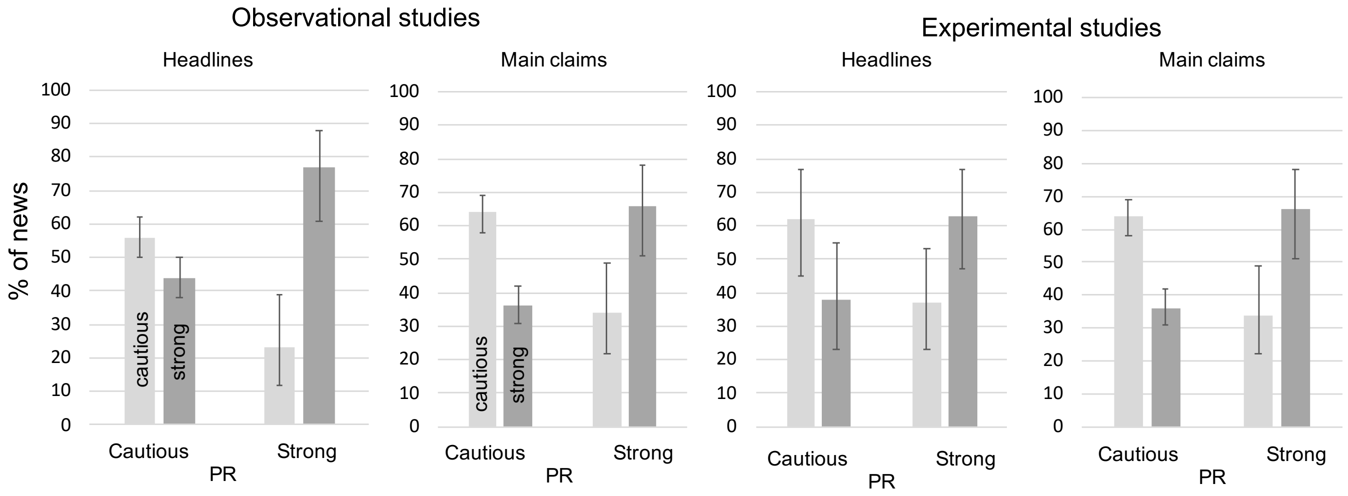

Figure S6. The proportion of news with cautious (light bars) or strong (dark bars) headlines or main claims depending on the cautiousness of press release headlines or claims, and separated by study design. These plots unpack the AT results for news content in Figure 2A of the main report (using GEE as in Figure 2). For observational studies, *cautious=aligned.* For Experimental studies, *strong=aligned.* Nevertheless, it is clear that the results are similar for both study designs: news headlines and claims appear to be sensitive to press release wording, but are not sensitive to study design *per se (*there were no significant interactions of the associations between news and press releases with study design). Error bars show 95% CI.

S7. News uptake for causal headlines and claims by study design.

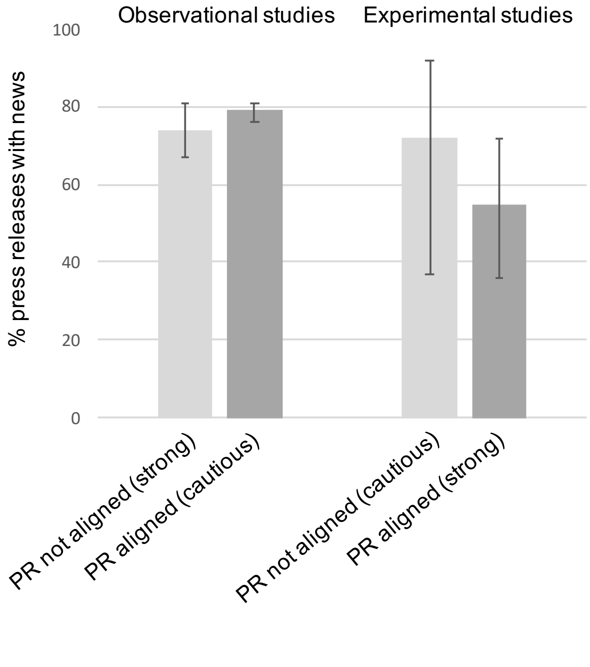

Figure S7. The proportion of press releases with news showed no significant sensitivity to whether the press release had aligned (light bars) or non-aligned (dark bars) headlines and claims for either study design. These plots unpack the AT results in Figure 2B of the main report. Error bars show 95% CI.

S8. Causality statements/caveats by study design.

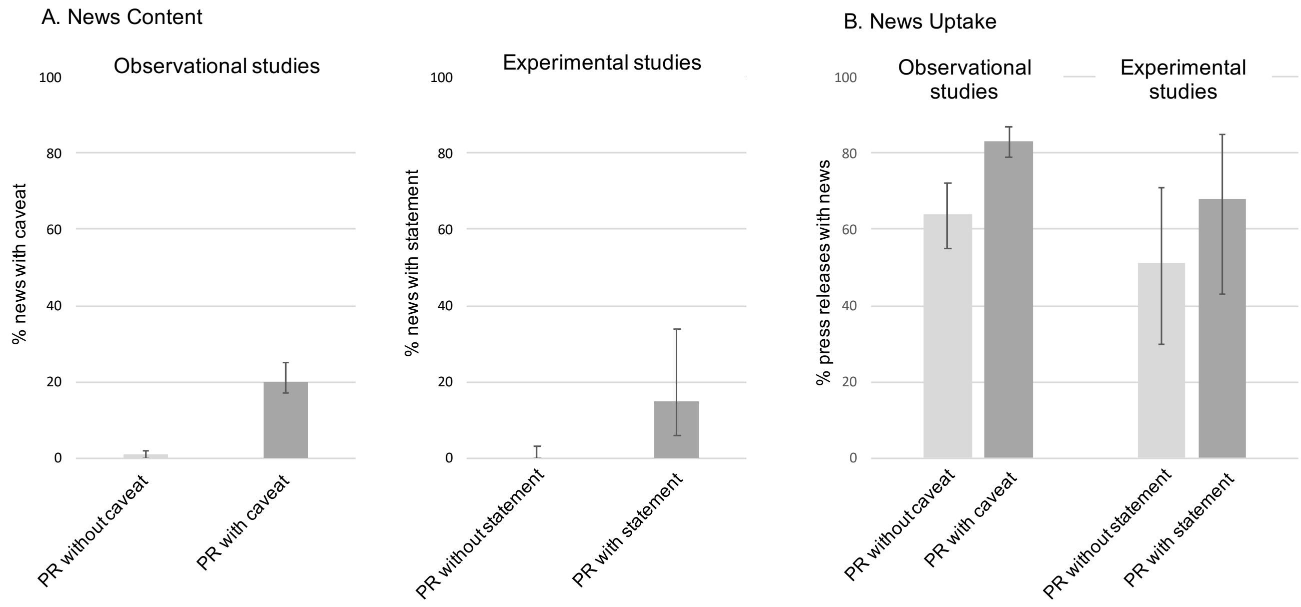

Figure S8. These plots unpack the AT results in Figure 4A,B of the main report. **A)** Caveats about causality almost never appeared in news unless they did in the press release, but their penetration to news from press releases was as good for explicit caveats about causality for observational research as for statements about causality for experimental research (rightmost bars in Panel A). **B)** When such caveats or statements occurred in press releases, news uptake was in fact higher. Error bars show 95% CI.
